# Campylobacter Antimicrobial Drug Resistance among Humans, Broiler Chickens, and Pigs, France

**DOI:** 10.3201/eid1302.060587

**Published:** 2007-02

**Authors:** Anne Gallay, Valérie Prouzet-Mauléon, Isabelle Kempf, Philippe Lehours, Leila Labadi, Christine Camou, Martine Denis, Henriette de Valk, Jean-Claude Desenclos, Francis Mégraud

**Affiliations:** * Institut de veille sanitaire, Saint Maurice, France; † Centre National de Référence des Campylobacter and Helicobacter, Bordeaux, France; ‡ Agence française de sécurité sanitaire des aliments (AFSSA), Ploufragan, France

**Keywords:** Campylobacter, surveillance, antimicrobial, resistance, research

## Abstract

We describe isolates from human Campylobacter infection in the French population and the isolates' antimicrobial drug resistance patterns since 1986 and compare the trends with those of isolates from broiler chickens and pigs from 1999 to 2004. Among 5,685 human Campylobacter isolates, 76.2% were C. jejuni, 17.2% C. coli, and 5.0% C. fetus. Resistance to nalidixic acid increased from 8.2% in 1990 to 26.3% in 2004 (p<10^-3^), and resistance to ampicillin was high over time. Nalidixic acid resistance was greater for C. coli (21.3%) than for C. jejuni (14.9%, p<10^-3^). C. jejuni resistance to ciprofloxacin in broilers decreased from 31.7% in 2002 to 9.0% in 2004 (p = 0.02). The patterns of resistance to quinolones and fluoroquinolones were similar between 1999 and 2004 in human and broiler isolates for C. jejuni. These results suggest a potential benefit of a regulation policy limiting use of antimicrobial drugs in food animals.

Campylobacter infections are, along with Salmonella infections, the most common cause of bacterial diarrhea in humans worldwide (*1**–**6*). A recent study on illness and death due to foodborne infections in France estimated an isolation rate of 27–37/100,000 persons/year for Campylobacter infection (*7*).

Campylobacter are part of normal enteric flora in animals (poultry, pigs, and cattle) and can be transmitted to humans through contaminated foods (*8*). Several studies identified chicken as the main source of infection (*9**,**10*). Most Campylobacter enteric infections are self-limited and do not require antimicrobial drug treatment. However, severe or long-lasting Campylobacter infections do occur and may justify antimicrobial drug therapy. Macrolides as first-line therapy and fluoroquinolones as alternative therapy are recommended (*2**,**11*). Resistance of Campylobacter to antimicrobial agents has increased substantially during the past 2 decades and has become a matter of concern in severe human Campylobacter infections (*12**–**14*). Combined studies in humans and poultry have implicated the use of fluoroquinolones in poultry in the emergence of drug resistance (*15**–**17*). As a consequence, in 2004 the US Food and Drug Administration withdrew the 1995/1996 approval for the new animal drug application to use enrofloxacin for prophylaxis treatment or growth promotion in poultry (*18*). Veterinary licensing of enrofloxacin in poultry was approved by the European Union (EU) in 1991, and in 1999 the EU recommended limiting the use of fluoroquinolones in poultry.

In this article, we describe characteristics of human Campylobacter isolates in France and trends of antimicrobial resistance in such isolates from 1986 to 2004. Trends of Campylobacter antimicrobial drug resistance in human isolates were compared with those of isolates from broiler chickens and pigs between 1999 and 2004.

## Materials and Methods

### National Surveillance System for Human Campylobacter Infections

Surveillance for Campylobacter infections in France is based on a network of laboratories that send their isolates to the National Reference Center for Campylobacter and Helicobacter (CNRCH). The network of participating laboratories, limited to hospital laboratories from 1986 to 2001, was complemented by private (which usually cared for outpatients) and additional hospital laboratories in 2002 to be more representative of the whole French territory (*19**–**21*). The network is currently composed of 325 private laboratories (9% of the 3,444 registered private laboratories in France), located in 90 of the 95 districts in mainland France, and 92 hospital laboratories (25% of the 409 registered hospital laboratories). Participating laboratories perform a systematic screening of Campylobacter in stools. Each isolate recovered is sent to CNRCH in a transport medium (medium for storage of bacteria, Bio-Rad, Marne La Coquette, France) with information on the type of specimen; date and district of isolation; patient's age, sex, and history of travel abroad; and eventual context of an outbreak.

On reception at CNRCH, isolates are tested for viability, confirmed as Campylobacter by standard phenotypic identification, and identified at the species level with phenotypic methods and real-time PCR to differentiate between C. jejuni, C. coli and C. fetus (*2**,**22*). The other species are identified by comparing their 16S rDNA sequences to those of DNA databases by using the BLAST program (*23*). Identification at the species level is considered correct when at least 99% identity occurs with only 1 species.

### Antimicrobial Drug Resistance Monitoring of Campylobacter in Humans

Campylobacter isolates from all species were evaluated for susceptibility to 7 antimicrobial drugs (nalidixic acid, ciprofloxacin [since 2000], erythromycin, amoxicillin, gentamicin, tetracycline, and doxycycline [since 2003]) by the agar diffusion method on Mueller-Hinton agar enriched with 5% sheep blood by using antibiotic disks, according to recommendations for Campylobacter of the Antibiogram Committee of the French Society for Microbiology (CA-SFM) (*24*). Since the hospital laboratory network set up in 1986 was extended to private laboratories in 2002, antimicrobial susceptibility trends were analyzed exclusively for hospital laboratory isolates between 1986 and 2004. Multidrug resistance was defined as resistance to >2 antimicrobial drugs.

### Antimicrobial Drug Resistance Monitoring in Broilers and Pigs

Surveillance of Campylobacter antimicrobial drug resistance was implemented in France in 1999 for broilers in conventional and free-range broiler farms and in 2000 for pigs as part of a surveillance program on resistance in sentinel bacteria (Escherichia coli and Enterococcus spp.) and zoonotic bacteria (Salmonella spp. and Campylobacter spp.) in animal products for human consumption. Thus, data collection began just after the ban of 4 antimicrobial growth promoters (bacitracin zinc, spiramycin, virginiamycin, and tylosin phosphate) by the European Community (EC) Council Regulation (No. 2821/98, December 1998). Conventional broiler flocks are characterized by an indoor rearing period of 6 weeks, and free-range broiler flocks have an indoor rearing period of 6 weeks followed by 6 additional weeks with access to an open-air area.

From 200 to 600 broiler cecal samples or pig fecal samples were collected each year in 10 broiler and 10 pig slaughterhouses representative of French production of these animals for human consumption (*25*). Strain isolation was performed in a central laboratory (Agence française de sécurité sanitaire des aliments [AFSSA], Ploufragan, France) for the first 2 years and then in district veterinary laboratories, except for antimicrobial susceptibility testing. After identifying isolates by using multiplex PCR (*26*), the MIC of ampicillin, nalidixic acid, enrofloxacin or ciprofloxacin, tetracycline, erythromycin, and gentamicin were determined by agar dilution. As for human isolates, susceptibility to antimicrobial drugs was categorized according to the 2004 statement of the CA-SFM (*24*). The study of antimicrobial resistance of Campylobacter from animal sources was supported by the French Ministry of Agriculture.

### Statistical Analysis

Differences between proportions and isolation rates were tested by χ^2^ and Fisher exact tests. Means were compared with Student and Fisher tests. Patterns of antimicrobial resistance were analyzed by 4-year increments from 1986 to 2004.

## Results

### Surveillance for Human Campylobacter Infections

From April 2002 to December 2004, CNRCH received 5,685 presumptive Campylobacter isolates. Among the 5,112 (89.9%) viable isolates, 3,896 (76.2%) were C. jejuni, 878 (17.2%) C. coli, 257 (5.0%) C. fetus, 21 (0.4%) C. lari, 40 (0.8%) Arcobacter butzleri, and 13 (0.25%) other species of Campylobacter. Seven strains (0.1%) were Helicobacter spp. A seasonal increase during the warmer months was noted and was more pronounced for C. jejuni.

**Figure 1:**
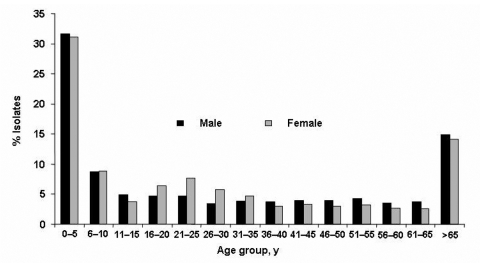
Distribution of Campylobacter isolates according to age and sex of patient, France, 2002–2004.

**Figure 2:**
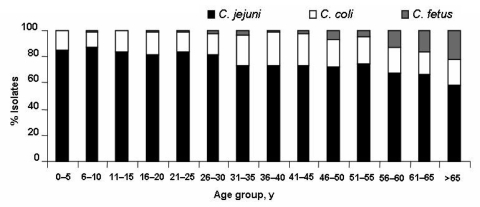
Distribution of human Campylobacter strains by species and patient age group, France, 2002–2004.

The median age of patients was 29.4 years (range 5 days–100 years). Thirteen (0.2%) were newborns (5–30 days), 258 (4.5%) infants (1–11 months), 1,907 (33.5%) children (1–10 years), 2,555 (44.9%) ages 11–65 years, and 767 (13.5%) >65 years (Figure 1). Isolation of Campylobacter was more frequent among male than female patients (male/female ratio = 1.2, p = 0.04), except for young adults (16–30 years), with a male/female ratio = 0.9 (p<10^-3^). The ratio of C. jejuni to C. coli varied between 4.5 and 7.2 in those <30 years of age and decreased thereafter. C. fetus was isolated among adults >30 years of age and peaked in the elderly (p<10^-3^, Figure 2).

Among the 5,620 isolates with a known clinical source, 5,253 (93.4%) were isolated from stools, 308 (5.5%) from blood, and 50 (0.9%) from other sites presumably seeded as a result of bacteremic infections. Both C. jejuni and C. coli were isolated essentially from stools, whereas 158 (63.5%) of 249 C. fetus isolates were from blood. Patients with blood isolates were older than those with stool isolates (median age 69 years vs. 19.3 years, p<10^-3^).

Travel history was available for 1,370 (24.1%) case-patients; 184 (3.2%) reported traveling outside France during the 2 weeks before onset of illness. The country of travel was specified for 169 (91.8%) case-patients (Africa, 98 persons; Asia; 26; Europe, 16; South America, 10; and other countries, 19).

### Antimicrobial Drug Resistance of Human Campylobacter Isolates

**Figure 3:**
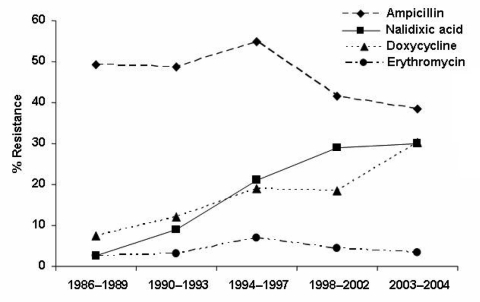
Resistance of human Campylobacter spp. isolates to antimicrobial agents, France, 1986–2004.

Resistance to nalidixic acid and tetracycline/doxycycline increased from 1986–1989 to 2002–2004 (p<10^-3^, Figure 3). Resistance to ampicillin, although frequent, decreased from 49.2% (1,027/2,087) in 1986–1989 to 42.4% (501/1,198) in 2002–2004 (p<10^-3^). Resistance to erythromycin remained low, and no isolate was resistant to gentamicin.

**Figure 4:**
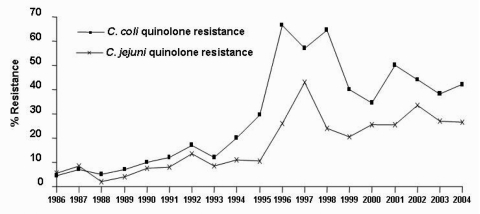
Quinolone resistance of human Campylobacter jejuni and C. coli; France, 1986–2004.

Nalidixic acid resistance increased from 8.2% (26/315) in 1990 to 26.3% (115/438) in 2004 (p<10^-3^). Resistance was greater for C. coli (21.3%) than C. jejuni (14.9%, p<10^-3^, Figure 4). Nalidixic acid resistance increased for C. jejuni >4-fold from 1995 to 1997 and for C. coli >3-fold from 1994 to 1996. Then, resistance decreased for both C. coli and C. jejuni in 1999 but remained higher than before 1995 (Figure 4). Ciprofloxacin resistance, tested since 2000, followed the same pattern (Table).

**Table:** Table. Resistance of *Campylobacter jejuni* and *C.* *coli* to ampicillin, erythromycin, tetracycline/doxycycline, and ciprofloxacin/enrofloxacin in humans, broilers, and pigs; France, 1999–2004

Antimicrobial agent/year	*Campylobacter jejuni*		*C. coli*		
	Humans, no. (%)	Broilers, no. (%)	Humans, no. (%)	Broilers, no. (%)	Pigs, no. (%)
Ampicillin					
1999	123 (42.3)	297 (22.6)	20 (70.0)	96 (29.2)	*
2000	189 (40.2)	67 (31.3)	35 (37.1)	35 (31.4)	317 (12.3)
2001	137 (47.4)	61 (13.1)	26 (53.8)	44 (25.0)	291 (11.0)
2002	184 (45.6)	41 (29.3)	43 (48.8)	64 (28.1)	101 (14.9)
2003	479 (46.1)	46 (34.8)	68 (28.1)	60 (30.0)	101 (17.8)
2004	438 (38.1)	32 (28.1)	88 (14.9)	74 (24.3)	67 (7.5)
Erythromycin					
1999	123 (0.8)	297 (0.3)	20 (10.0)	96 (31.3)	*
2000	187 (3.2)	67 (3.0)	35 (8.6)	36 (11.1)	317 (65.3)
2001	136 (3.7)	61 (4.9)	26 (7.7)	44 (4.5)	289 (49.1)
2002	184 (2.7)	40 (5.0)	44 (9.0)	64 (17.2)	101 (58.4)
2003	478 (0.6)	46 (4.3)	68 (7.3)	61 (31.1)	97 (78.4)
2004	437 (1.4)	32 (0.0)	88 (12.5)	74 (17.6)	67 (43.3)
Tetracycline/ doxycycline					
1999	118 (23.7)	297 (56.6)	6 (25.0)	96 (69.8)	*
2000	188 (12.8)	67 (55.2)	35 (14.3)	35 (60.0)	317 (82.6)
2001	137 (9.5)	61 (65.6)	26 (26.9)	45 (80.0)	289 (88.9)
2002	184 (22.8)	41 (67.5)	43 (41.8)	64 (84.4)	101 (86.1)
2003	479 (26.7)	46 (60.9)	68 (63.2)	61 (96.7)	97 (95.9)
2004	438 (28.8)	32 (40.6)	88 (53.4)	74 (71.6)	67 (61.2)
Ciprofloxacin/ enrofloxacin					
1999	†	297 (16.8)	†	96 (39.6)	*
2000	185 (23.8)	68 (23.5)	35 (31.4)	35 (28.6)	316 (12.3)
2001	137 (21.9)	61 (29.5)	26 (34.6)	45 (37.8)	292 (12.3)
2002	184 (31.7)	41 (31.7)	44 (43.2)	63 (41.3)	101 (21.8)
2003	479 (25.9)	45 (13.3)	68 (38.2)	61 (41.0)	99 (24.2)
2004	438 (25.3)	32 (9.4)	88 (42.0)	74 (32.4)	67 (26.9)

*Strains isolated from poultry in 1999 and 2000 were tested with enrofloxacin; strains isolated between 2001 and 2004 were tested with ciprofloxacin; all pig strains were tested with ciprofloxacin.

†Human strains were tested for ciprofloxacin since 2000.

Fifty-eight percent of Campylobacter isolates were resistant to >1 drug, 34.7% to >2 drugs, and 20.0% to >3 drugs. The most common multidrug resistance (>2 drugs) patterns included resistance to nalidixic acid or ciprofloxacin, to doxycycline, and to ampicillin.

Among the case-patients £15 years of age, 28.0% (618/2,207) had a Campylobacter strain resistant to nalidixic acid compared with 37.6% (1,029/2,736) of the case-patients >15 years of age (p<10^-3^). The proportion of resistance to ciprofloxacin did not vary according to age (27.3% of case-patients £15 years and 27.9% >15 years). For ampicillin, 41.9% (925/2,207) of case-patients £15 years had a resistant strain compared with 37.3% (1,024/2,736) of the case-patients >15 years (p = 0.001).

Of the case-patients who traveled abroad, for which strain resistance was available, 40.3% (67/166) had a strain resistant to ciprofloxacin, compared with 27.0% (294/1,090) of case-patients who did not travel abroad (p<10^-3^). For nalidixic acid, 42% (70/166) of case-patients who traveled abroad compared with 34.7% (378/1,090) of case-patients who did not had a resistant strain (p = 0.06). Resistance to ampicillin was present for 28.3% (47/166) who had traveled abroad compared with 31.1% (339/1,090) for those who had not (p = 0.01).

### Antimicrobial Resistance in Broilers and Pigs

Between 1999 and 2004, a total of 544 C. jejuni and 374 C. coli isolates were recovered from poultry, and 871 C. coli were recovered from pigs by the antibiotic resistance surveillance system. Among the broiler isolates, the proportion of C. jejuni from animals raised in standard and export production facilities gradually decreased from 83.5% (279/334) in 1999 to 43% (28/65) in 2004 (p<10^-3^), while the proportion of C. jejuni decreased from 32% (18/57) to 10% (4/40) in the free-range production facilities (p = 0.01).

Campylobacter isolates were inconstantly sensitive to ampicillin, and a high proportion of isolates resistant to tetracycline was recorded in poultry and pigs, but all strains remained sensitive to gentamicin (Table). Isolates from pigs were less frequently resistant to ampicillin but more often resistant to tetracycline. For erythromycin, resistance was rare among C. jejuni strains (1.8%), but much more frequent for C. coli (21.1% of broiler isolates and 58.9% of pig isolates, p<10^-3^).

**Figure 5:**
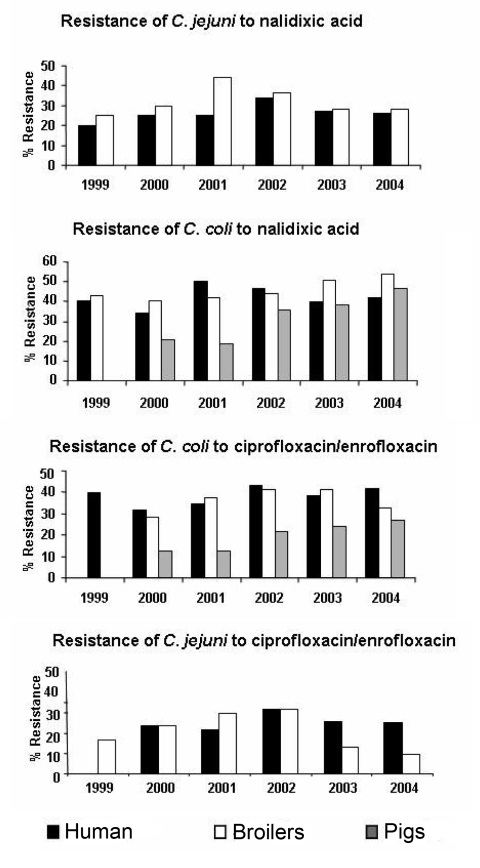
Resistance of Campylobacter jejuni and C. coli to nalidixic acid and ciprofloxacin in humans, broilers, and pigs, France, 1999–2004.

In broilers, C. coli strains were more often resistant to ciprofloxacin (37.4%, 140/374) than were C. jejuni strains (19.5%, 106/544), p<10^-3^) (Table, Figure 5). C. coli resistance to ciprofloxacin increased in pigs from 12.3% (39/316) in 2000 to 26.9% (18/67) in 2004 (p = 0.002). For C. jejuni in broilers, after an increase in resistance from 16.8% (50/297) in 1999 to 31.7% (13/41) in 2002 (p = 0.02), resistance to ciprofloxacin decreased to 9.0% (3/32) in 2004 (p = 0.02) (Table, Figure 5). Similar trends were observed for quinolone resistance. The trend of resistance to nalidixic acid and ciprofloxacin was similar for C. jejuni isolated from humans and broilers between 1999 and 2004 (Table, Figure 5).

## Discussion

Our surveillance of Campylobacter isolates in France indicates some differences with findings from other western countries, i.e., a greater proportion of C. coli (17.0%). The epidemiologic characteristics of Campylobacter infections were, however, similar. Campylobacter is predominant in the summer (*27*), the isolation rate was much greater in children <5 years of age, and C. jejuni was predominant. The rate of resistance to certain antimicrobial drugs increased substantially from 1990 to 2004, and the proportion of resistant isolates varied according to Campylobacter sp. Resistance to quinolones was greater for C. coli (21.3%) than for C. jejuni (14.9%). Quinolone and fluoroquinolone resistance for C. coli was high in broilers and pigs. Resistance to quinolone and fluoroquinolone for C. jejuni had the same pattern over time in broilers and human isolates.

The proportion of C. coli was higher in France (17%) than in the United States (<1%) or Belgium (11%) (*2**,**28*). Methods for characterization of the species vary by country and by laboratory within a country (*29**,**30*). In France, CNRCH routinely characterizes the species using a combination of phenotypic and molecular methods (specific PCR) with verification of discrepant results (*22*). In some other countries, Campylobacter are not routinely characterized at the species level and could be incorrectly identified as C. jejuni or Campylobacter spp. This may account for an underestimation of species other than C. jejuni in some countries and therefore some distortion of the proportion of antimicrobial drug resistance by species. In France, the high proportion of C. coli isolates is probably real, as an increasing proportion of C. coli is colonizing broilers (*31*). This trend may be related to the use of different isolation and identification methods, to a recent increase in the ratio of C. coli to C. jejuni, or both. The ban of antimicrobial growth promoters and of animal protein–based feed may have influenced the digestive bacterial flora equilibrium of chickens. Udayamputhoor et al. showed that the ceca of birds that receive plant protein–based feed are less likely to be colonized with C. jejuni than the ceca of birds that receive other types of feed (*32*). Another hypothesis is that because 60% of pigs are colonized by C. coli in France, the proximity of pig and poultry farms in the main producing regions may result in cross-contamination. However, this explanation is unlikely because C. coli strains isolated from broilers and pigs had different antimicrobial resistance patterns, and C. coli poultry strains clustered separately from those of porcine origin (*33*). Nonetheless, strains may undergo different selection pressures.

Resistance to ampicillin is of clinical interest because this drug may be used for the treatment of severe Campylobacter infections. The proportion of resistance to ampicillin was higher among patients who did not travel than among patients who did and in children <15 years. In addition, resistance of Campylobacter isolates in humans did not follow the same patterns over time as resistance in broiler and porcine isolates. These results suggest that resistance to ampicillin is more frequently domestically acquired and may be related to the use of ampicillin in human therapy because ampicillin is widely prescribed for infections in children.

Nalidixic acid resistance increased 5-fold from 1990 (5.3%) to 2004 (26.3%), consistent with trends observed in other countries (*16**,**17*). The use of fluoroquinolones in animal feed was approved in Europe in 1990. Studies have shown the development of ciprofloxacin-resistant Campylobacter in treated chickens and the spread of ciprofloxacin-resistant Campylobacter from animal food sources to humans (*17**,**34**,**35*). Australia, where fluoroquinolones have never been licensed for use in food-producing animals, did not experience fluoroquinolone resistance in human Campylobacter isolates (*36*).

The high proportions of resistance to nalidixic acid and ciprofloxacin in broilers and pigs are consistent with the findings of Desmonts et al. in France (*31*). In this study, quinolone and fluoroquinolone resistance increased between 1992–1996 and 2001–2002 among isolates from standard chicken flocks, while resistance remained low for free-range flock isolates. In France, antimicrobial growth promoters have never been authorized in the production of free-range chickens, contrary to standard methods of production of chicken flocks, and antimicrobial therapy is limited (*31*).

From 2002 to 2004, ciprofloxacin resistance dropped substantially in C. jejuni isolated from broilers; nalidixic acid resistance decreased as well, although not significantly. The decrease in broilers may be related to the restriction in the use of fluoroquinolones in animal feed after the 1999 EU recommendation. Similarly, in Denmark, resistance to macrolides of C. coli declined after the prophylactic and growth-promoting use of macrolides was banned (*37*). However, the decrease in ciprofloxacin resistance occurred 2–3 years after the EU recommendation, which suggests that EU recommendations were not followed immediately by application or, alternatively, that the effect of the restriction in the use of fluoroquinolones in animal feed is not immediate. Unfortunately, no resistance data in broilers and pigs were available before 1999, which is a limitation to interpret recent trends in relation to the EU recommendations. According to the French food security agency (AFSSA), global sales of antimicrobial agents decreased consistently from 2001 through 2002, but information on species-specific consumption was not available (*38*). Specific data from veterinary prescriptions and livestock consumption are necessary to quantify the amount of antimicrobial agents consumed by animals.

C. jejuni nalidixic acid and ciprofloxacin resistance decreased concomitantly in humans and broilers from 2002 to 2004. Because the decrease was less pronounced in humans than in broilers, a longer period is needed to detect an effect of the restriction in the use of antimicrobial agents in animal feeds or resistance may be also related to other exposure. Fluoroquinolones are the first drugs of choice for the empiric treatment of human diarrhea or prophylactic treatment associated with travel in France and may be responsible for a part of resistance in humans (*39*). However, >80% of patients infected with a ciprofloxacin-resistant strain did not travel to a foreign country before onset of illness, which indicates that a substantial proportion of fluoroquinolone resistance was domestically acquired (*40*). The resistance rate to ciprofloxacin was not higher in adults compared with children, as could be expected if treatment of cases was contributing to resistance (*39*), because fluoroquinolone treatment is not used in children <15 years of age.

In contrast to C. jejuni, we observed no decrease in quinolone and fluoroquinolone resistance in C. coli in pigs, broilers, or human isolates. The use of these antimicrobial agents in pigs may not have changed, and a part of human Campylobacter coli infection may be related to other sources. Alternatively, unknown mechanisms could be implicated in C. coli resistance, such as a high number of point mutations.

Our study has several limitations. Laboratories participate voluntarily in the surveillance network, which may result in a selection of strains that is not representative of the general population. However, all districts of mainland France were included. In addition, this limitation, if it exists, may not affect antimicrobial drug resistance because participating laboratories routinely screen stools for Campylobacter and send all their isolates to CNRCH. Since the laboratory network was extended to private and additional hospitals laboratories in 2002, this may have had an effect on the trends in resistance. However, trends of human antimicrobial susceptibility were analyzed exclusively among strains from hospital laboratories from 1986 to 2004. In addition, characteristics of Campylobacter isolates sent to CNRCH did not change for age, sex, seasonality, and species after the network extension in 2002 (20,21). Comparison of human and animal data was not based on a continuum between human isolates and contaminated food consumption (isolates from retail chicken). However, broiler chicken and pig data were representative of French livestock and were consistent with those of another recent survey done in France (*31*).

The extension of the surveillance of human Campylobacter allowed the epidemiologic characteristics of Campylobacter infections that occurred in the general French population to be better understood. Campylobacter resistance to antimicrobial agentss increased to a high level among humans in France from 1990 through 2004. Comparison of antimicrobial resistance patterns in humans, broilers, and pigs from 1999 to 2004 showed similar patterns of quinolone and fluoroquinolone resistance for C. jejuni isolates from broilers and humans. These results suggest that a limitation of the use of fluoroquinolones in broilers may reduce fluoroquinolone resistance of Campylobacter in humans. Other studies, however, are needed to further quantify the effect of restricted use of antimicrobial drugs in animals on bacterial resistance in human isolates. Ongoing national surveillance of Campylobacter in humans, livestock, and animal feeds at the retail level and antimicrobial susceptibility testing are necessary to evaluate the effects of implementing European policies. Further research is also needed to better understand the relationship between antimicrobial use in animals and humans and bacterial resistance in humans.
